# Supplementary material for: Evaluation of Reference Genes for Normalizing RT-qPCR and Analysis of the Expression Patterns of WRKY1 Transcription Factor and Rhynchophylline Biosynthesis-Related Genes in Uncaria rhynchophylla
Source: Int J Mol Sci. 2023 Nov 15;24(22):16330. doi: 10.3390/ijms242216330 (PMC10671239; doi:10.3390/ijms242216330)
Supplement: Supplementary file 1 [file ijms-24-16330-s001.zip › Supplement dataset S1.pdf]

Additional files

Supplementary Table S1. Expression stability values of 10 candidate genes calculated using the RefFinder in *U. rhynchophylla*

Rank	MeJA	ETH	Low temperature	Total
1	*PP2A*	*SAM*	*SAM*	*SAM*
	1.189	*1.495*	*1.50*	1
2	*ACT6*	*cdc73*	*cdc 73*	*cdc 73*
	2	*2.213*	*2.632*	2.213
3	*cdc73*	*ACT6*	*PP2A*	*ACT6*
	3.464	*3*	*2.931*	2.632
4	*SAM*	*CYP*	*CYP*	*PP2A*
	3.464	*4.356*	*3.13*	4.356
5	*18S*	*PP2A*	*18S*	*CYP*
	5	*4.427*	*4.141*	4.356
6	*CYP*	*PAL*	*PAL*	*PAL*
	6	*4.472*	*4.606*	5.958
7	*GAPDH*	*EF-1β*	*GAPDH*	*EF-1β*
	7.483	*5.292*	*6.402*	6.435
8	*PAL*	*GAPDH*	*ACT6*	*DAPDH*
	7.969	*7.483*	*7.737*	8.239
9	*EF-1β*	*TUA*	*TUA*	*18S*
	8.452	*9*	*9*	8.739
10	*TUA*	*18S*	*EF-1β*	*TUA*
	10	*10*	*10*	10

Supplementary Table S2. Primer sequences of genes of interest in *U. rhynchophylla*

Gene abbreviation	Gene Name	Primer Sequence 5′-3′ (F/R)
WRKY1	WRKY1 transcription factor	GACAGTGACATATCCAAGAGACAGA<br>GCCAGCGATAGCCATCATTTAC
SGD	Strictosidine-β-D-glucosidase	GTGCTTATGGGGACTTAATGATTGATC<br>CTCTTGGAGAGTAATTGGTTCTGAGC
STR	Strictosidine synthase	CCATCTTCGCTAAGAAATCAAACCAAA<br>GTTGCTGATTTTTCCTCACTTTTTGGG
TDC	Tryptophan decarboxylase	CGTTACAAACTGAACCCAAACCAG<br>GGTTTCTTGTGGAAGATTTCCACCTC
SLS	Secologanin synthase	CTCATAGGTGCATTGATGTGGTTG<br>ACTGGTCCGTACTTGCTTCTC

TSA	Tryptophan synthase alpha chain	AGAGAGGCTTTGCACGTAGTCTTC<br>TCAGACAGTTGCCACCGCTTTC
AnPRT	Anthranilate phosphoribosyltransferase	GCTTGTGGAAGTGCTGATGTT<br>GCTGGATGGTAGATTGGAGACA
7-DLH	7-deoxyloganic acid 7-hydroxylase	TGCCGACGAAGAGGAACAA<br>ATGCCCAAGAAGTTATCACCAGTA
7-DLS	7-deoxyloganetic acid synthase	GCTGGTCTTCTTCTCCTTCTGATAA<br>ACTGGTCCGTACTTGCTTCTC
7-DLGT	7-deoxyloganetic acid glucosyltransferase	CCGCAACAGAGCAGTCAATG<br>CCTCATCACCCACAGAAACCT
PRAI	Phosphoribosylanthranilate isomerase	TTGGTGTGTTTGTAGACGATAATGC<br>GAGTTCAGTAGGCTTCCATCTTCA
G8H	Geraniol 8-hydroxylase	GCTGAGGTGACTGAACTATTAGG<br>GTGTCCGCTGGTCAATTATCT
8-HGO	8-hydroxygeraniol dehydrogenase	CTCTGCTGTTCATCCTCTCCTT<br>CCAATGCCACTTCCACCTAATATC
GES	Geraniol synthase	AGACACAGAAGGATGGCAAGG<br>ACCACCGAGTAATCTCAGACAAC
AS	Anthranilate synthase	CGGAATCAATCGGTTGGAGTCT<br>AACACTGCCTTCTCGTAGTCTTC

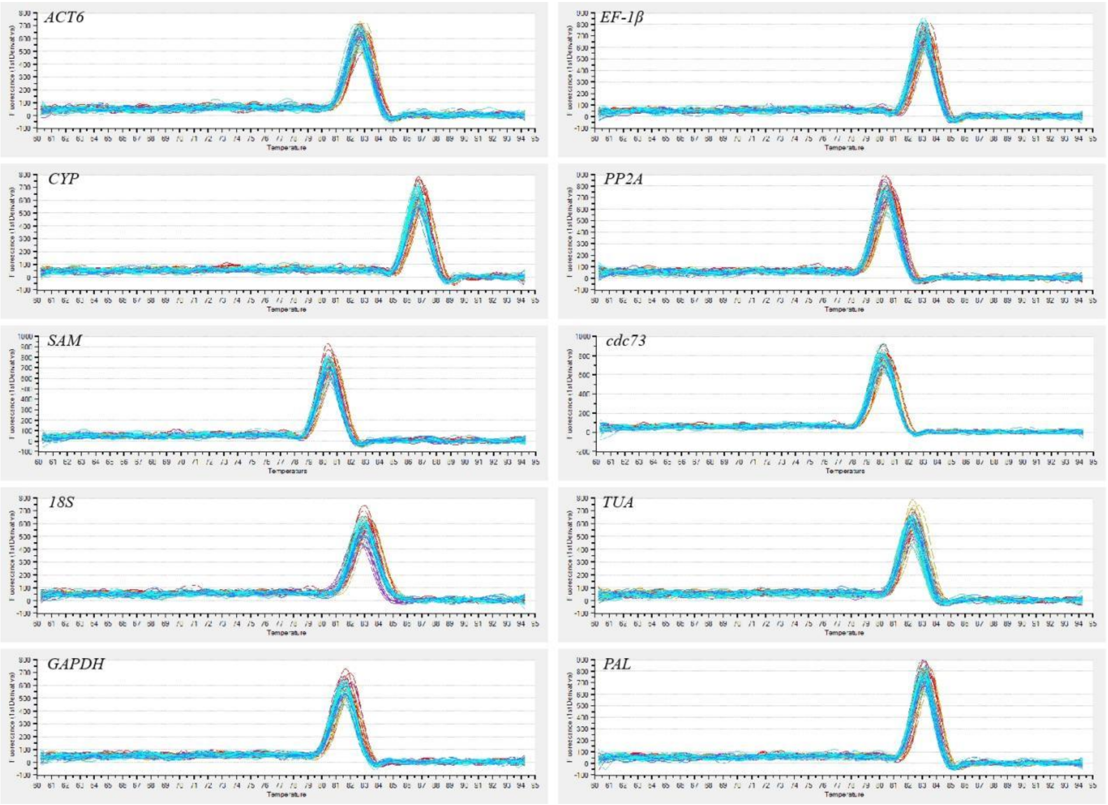

Supplementary Figure S1. RT- qPCR melting curves of ten reference genes.

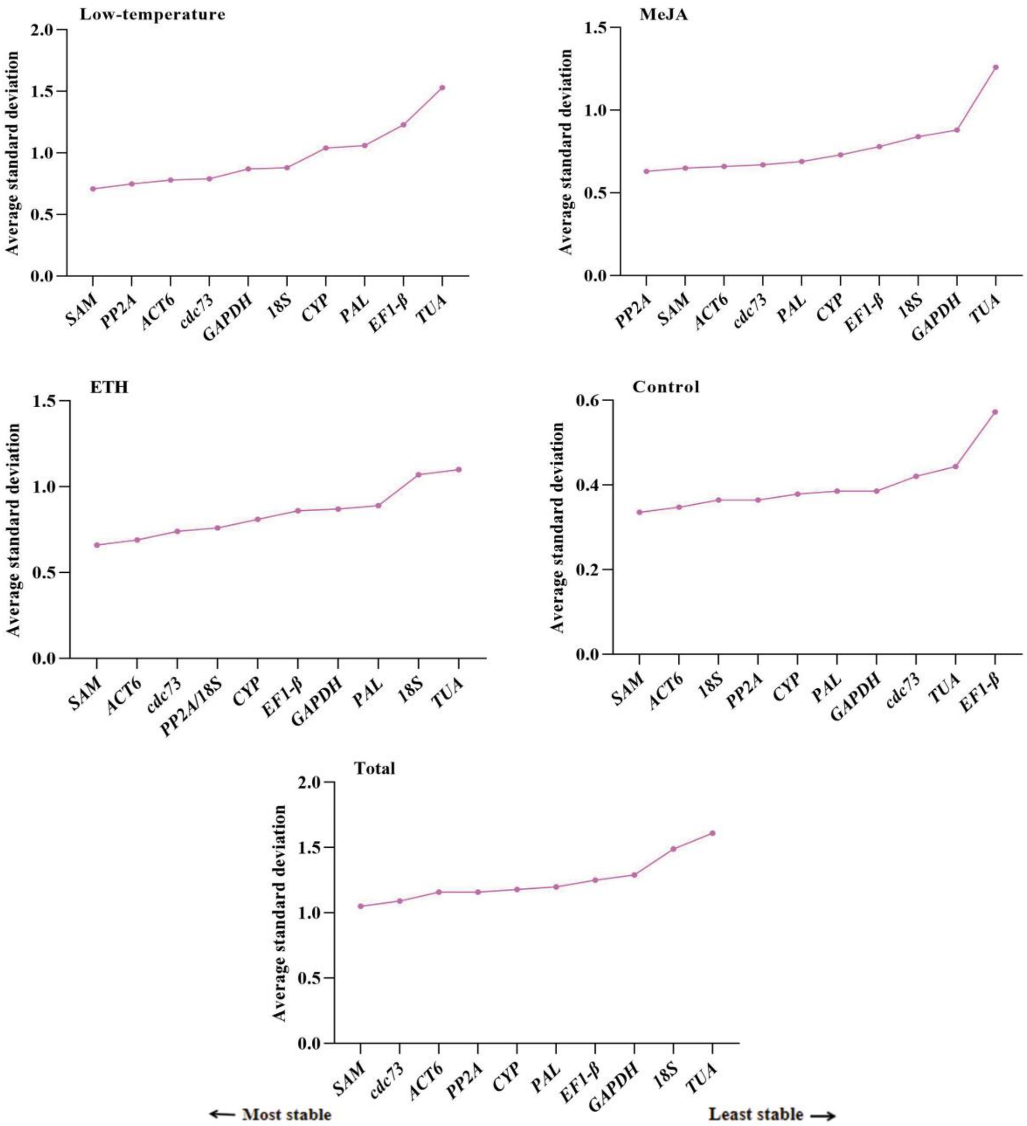

Supplementary Figure S2. Average standard stability deviation (SD) of the ten candidate reference gene using Delta Ct algorithm.

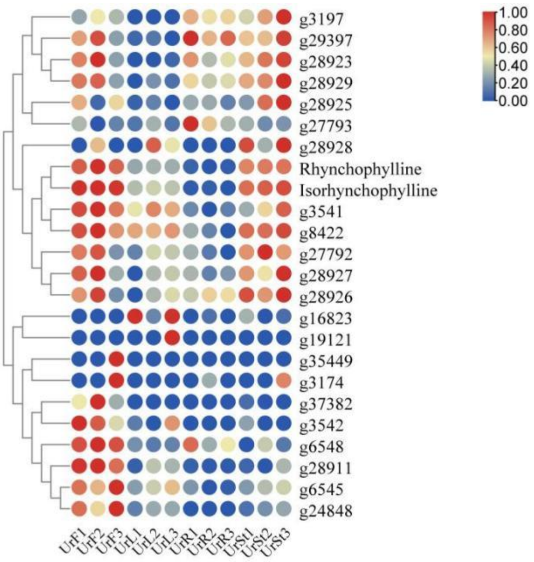

Supplementary Figure S3. Screening of strictosidine synthase genes (*STR*) heatmap. Rhynchophylline (RIN) and isorhynchophylline (IRN) are the content.

Additional files list

Supplementary Table S1. Expression stability values of 10 candidate genes calculated using the RefFinder in *U. rhynchophylla*

Supplementary Table S2. Primer sequences of genes of interest in *U. rhynchophylla*

Supplementary Figure S1. RT- qPCR melting curves of ten reference genes.

Supplementary Figure S2. Average standard stability deviation (SD) of the ten candidate reference gene using Delta Ct algorithm.

Supplementary Figure S3. Screening of strictosidine synthase genes heatmap. Rhynchophylline (RIN) and isorhynchophylline (IRN) are the content.
