# Supplementary material for: Evaluation of Reference Genes for Normalizing RT-qPCR and Analysis of the Expression Patterns of WRKY1 Transcription Factor and Rhynchophylline Biosynthesis-Related Genes in Uncaria rhynchophylla
Source: Int J Mol Sci. 2023 Nov 15;24(22):16330. doi: 10.3390/ijms242216330 (PMC10671239; doi:10.3390/ijms242216330)
Supplement: Supplementary file 1 [file ijms-24-16330-s001.zip › Supplement dataset S2.pdf]

Supplement dataset 2

>g36705.t1 Name:Geraniol synthase gene=g36705 CDS=1-1764
ATGGCTTGCATGAGCAAAGTTTCCTCACTATCAAAATCTTGGGCAGTTTTTG
ATGTCCCTCGTAGAGATCTTCCTAGGGCGACTCATTCATGGCTAAAAAGGC
AATTCAACTTCAAAACTTCACGCATTTGCATGTGCATGCCATCACAAACTTT
GAACCAGCATATAGCAACGCGACTGATCACAGATTATGAATCTTTGCTCAAA
TATTTGCGTCAACCTCTGGTGCTTCTACACGAAGTTGATGACAAAACCAAG
AGGAAGGAATTGTTAGAAAGAACAAGAAGGAAGCTGAGATCAACTTCAG
AACCTTTGGAGGCCATGAAATTGATAGACACACTTCAGCGACTGGGAATAG
CATACCATTTAGAAGATGATACGAATGCTATATTGTCAAGGTTTTCATCAGAT
GGCCAATATAATGAAGATCTCCTTACTGCTGCACTTCGTTTTCGATTGCTAA
GACATAATGGTCATAAAATTAGTCCTGAAATATTTCAAAACTTCAAAGACCA
TAACGGAAAATTTAAAGACTCCTTGCAAGAAGACACACTAGGGTTGTTGA
GCTTGTATGAAGCCTCACATTTGGGAGCAACTGGAGAAGACATATTGTTAG
AGGCCATGGAGTTCAGTGAAGCCCACCTTAAACAATCACTTTCCAAATTAG
AACCCCAACTTGCTACAAACGTTTCTCAGGCCTTGGACCTGCCAAGACAC
AGAAGGATGGCAAGGCTAGAAGCCAGAAGGTATATTGAAGAATATGATAAC
GAACGTGGCCACGATCCAGCCCTCCTCGAACTAGCCAAATTGGACTATAAC
AAAGTCCAGTCACTGCATCAAATGGAGTTGTCTGAGATTACTCGGTGGTGG
AAACAATTAGGTCTTGTAGATAAGCTGACTTTTGCTCGAGATCGGCCACTC
GAATGTTTTCTCTGGACCGTTGGAATACTTCCAGAACCGAAGTATTCAAGC
TGCAGAATTGAGTTAGCCAAGACTATTGCCATTTTATTGGTGATTGATGATAT
TTTTGATACTCAAGGGACCATTAATGAGCTTGTTCTCTTTACCAGTGCGATT
CGAAGATGGGATCTTGAAGCAATAGATGAGCTACCTGAATACATGAAAATA
TGTTACATGGCATTGTACAATACTACTAATGAAATCTGCTACAGGGTCCTCA
AGGAAAAGGGTTGGAGTGTCCTCCCATACCTAAAATCAACGTGGATAGATA
TGATTGAGGGGTTCATGCTGGAAGCAAGCTGGTATAACAATGGACAAGTAC
CAAACATGGAAGATTATGTAGAAAATGGTGTTACAACAGCAGGAGCCT
ACATGGCCTTGGTGCATCTATTCTTTCTTATTGGGCAAGGAGTTACAGAGGA
CAATGTAAAATTGTTGATGAAACCCTATCCAAAGCTCTTTTCCTGCTCGGGG
AGAATTCTTCGACTTTGGGATGATTTGGGAACTGCTAAGGAAGAGCAAGA
AAGAGGTGATCTTGCATCTAGCATACAATTGTACATGAGAGAGAACAACAT
AACTTCTGATGAAGAAGGCCGAAAAGGCATCTTGCAGCTCATAAACAACCT
ATGGAAGGACCTCAACTATGAATTAGTGTCTCGAAATGAATTGCCACTTGC
CATCATCAAAGCTGCATTCAACATGGCCAGAGCTTCTCAAGTTGTGTATCA
GCATGAGGAAGACAGCTACTTTTCCAGCGTTGATAATTATGTACAGGCTTTG
TTTTTCACTCCTTTAAGTAATTGA

>g4952.t1 Name:Geraniol 8-hydroxylase gene=g4952 CDS=1-1041
ATGACTATCAATTTCCAATTCCCTCATAAACTAAGCAGTTTCTTAAACAATG
ATGATCGCTTTCTTGTATTTCCAACACTGATCATCATTTTGATAGCAACATGG
TTTGCCTGGATACTTGTCAAATTAGCAAGGGGTCAAAAACCACTGCCACCA

GGTCCATGGGGTCTGCCTGTGGTAGGAAACCTTCCATTTCTTGATCCTGAA
CTCCACTCATACTTTGCTGGCCTAGCTCAATCCTATGGCCCTATCTTTAAACT
CAGGCTTGGTGGGAAGTTCAACATCGTGATTACTTCACCTGCGGTGGCAAG
GGAGGTGCTCAAAGACCAGGATGTAACCTTTGCCAACCGTGATGTCCCAGT
GGTGGTCACAGCCATGGAATATGGTGGCCATGACATAGTTTTTACACCATAC
GGTGCCGAGTGGCGGATGCTGAGGAAGGTTTGTGTTAGGGACATGCTTGG
CCATGCCAACCTTGATGCAGTCTACTCTTACCGAAGACAAGAAATCCGTAG
TACTATAAAACATTTGTATAGTCAAAAAGGATCTCCTGTTAATGTGGGTGAA
GAGATGTTCTTGAATGTACTCAATGTGATCACAAATATGTTATGGGGTGGTA
CTATTCAGGGGAAAGCAAGGAGTAACATTGGAGCTGAATTCAGGCAAGTA
GTTGCTGAGGTGACTGAACTATTAGGGAAGCCTAATGTATCTGATTTCTTTC
CACGGTTGGCAAAGTGGGATTTGCAAGGCGCCAAGAAGCAGATGAAAGT
AGTGACGTCCAAAATTGAGCAGATTTTTGACAAGATAATTGACCAGCGGAC
ACAAATAGATGGACAGAAAGGGATTAGAAGAGGAAATGGGAGCACGGGG
AGCAAAGACTTTTTGCAGGTTCTATTGCAGCTGAAAGATACTGGCGATGCC
AAAACACCTCTAACCATGAACCATGTCAAGGCTTTGCTTCTGGCATGTTTTA
CTACTACTACCTCTTTCATATACTACAAAATGTCAATTTATCTCATTGCCATTT
CACAAATAATCTAG

>g27625.t1 Name:8-hydroxygeraniol dehydrogenase gene=g27625 CDS=1-1083
ATGGCGAAATCAGCTGAAGGAGAGCACCCCCTCAAGGCTTTTGGTTGGGC
TGCCAGAGACGCGTCTGGTGTTCTATCTCCGTTTAGTTTCTCTAGGAGGGC
AACTGGAGAAAAAGATGTTCAAATCAAAATATTGTATTGTGGAATATGTCAC
TCTGATCTTCACTGTGCTAAGAATGAATGGGGCTCCACTAAATATCCTATTG
TTCCTGGGCATGAGATTGTTGGTGTGGTGACAGATGTAGGCAGCAACGCAA
CCAAGTTCAAAATTGGAGACAAGGTTGGTGTGGGATGCATGGTTGGATCAT
GCCTCTCCTGTGAGAACTGTTCAAACAATCTTGAAAATTACTGCAACAAAG
TCATTGCAACCTACAATTCAACCTATCACGATCAAACGCCCACATATGGGGG
CTATTCTGACATTTTGGTTGCTAATGAACATTTTGTAGTCCGAATTCCTGAA
AATATGCCACTTGATGCTGGTGCTCCTCTACTATGTGCTGGGATCACAACCT
ATAGCCCCCTGAGGTATTTTGGACTGGACAAACCTGTTATGCATATTGGTGT
TGTAGGCCTCGGTGGATTGGGACATATGGCTGTGAAGTTTGCCAAGGCATT
TGGGGTTAAGGTGACAGTTATTAGCACATCTTTGCATAAGAAGGAGGAAGC
CCTTGAACATCTTGGTGCTGATTCATTTTTAGCCAGCACTGATAAAGATCAG
ATGCAGGCTGCTATGGAGAGTATGGATGGTATCCTTGACACTGTCTCTGC
TGTTCATCCTCTCCTTCCATTAATTAGTCTCTTGAAGTCTCATGGAAAGCTT
GTTATCGTTGGCGCACCGGATAGGCCACTTGAGCTGCCAGTCTTTCCTATGC
TTGCAGGAAGAAAGATATTAGGTGGAAGTGGCATTGGGGGAATGAAGGAG
ACACAAGAAATGCTTGATTTTTCAGCAGAACATAACATAGTTGCTGATATTG
AGGTCATTTCGATGGATTATGTCAATACTGCCATGGAGCGGCTAGCCAAAGC
TGATGTCAAGTATAGATTTGTAATTGATGTTGGTAACACTCTAAAAGCTGCT
TAA

>g24241.t1 Name: 7-deoxyloganetic acid synthase gene=g24241 CDS=1-1179

ATGGTAATGAAGTGGACTTTGAAGTCACTAAACCCCGTGACCATTGCTGTC
TCAGCTGGTCTTCTTCTCCTTCTGATAACTTTAATCAAGCGGCGGAGTGGTT
CTTCTAAAAGAAGACCACCAGGCCCACCCGGATGGCCGATTTTTGGGAACA
TGTTCGACCTTGGGGACTTGCCACACCAAACTTTATACAGGATGAGAAGCA
AGTACGGACCAGTTGTTTGGCTGCAGCTGGGATCAATCAACACCATGGTTG
TACAGAATGCTGTTGCTGCTTCTGAACTATTCAAGAAGCACGATGTCCCATT
TGCTGACCGGAAGGTGCCTGATACCCTCACGGCCTTCAACTTCAACCAGGG
TTCGTTGGGCATGAACACCTTTGGAGGCCATTGGCGTGTGCTCAGGCGTCT
CTGCTCCATGGAGTTCCTCGTTAACAAAAGAATGAATGAGACCACTGACCT
AAGGAGGCGAATTGAGGACAACATGATAAGCTGGATTGTGGACGAGTCTA
ACTCTTCAAAAGCTCAAGGAGGAAATGGGGCGGTGCAACTCAGCCGCTT
GCTTTTTCTCATGGCATTCAACCTGGTTGGCAACCTTATGCTGTCGAGGGAT
TTGTTGGACAACAAGGACCCTGAAAGTCGGGAGTTTTTTGATTCCATGAAC
GAGATCCTGGAGTTGGCTGGAACTCCTAACATAGCAGATTTTCTACCGTTCC
TGAAAAGGCTTGACCCCTTGGGCATGAAGAAGAGAATGATAGCCAACATG
ACGAGAACCATGAGAATTTCTTCCAAATTTGTGCAGGAAAGACTTGACAA
CAGGAAAGCTGGCAGTGTTAATGACAAGAAAGACTTCTTGGATGTCATGTT
GGAGTACCAAGGTGATGGTAAGGATGGTCCTGACAGATTCACAGAGCAAC
ATGTCAACATTGTTATCATGGAAATGTTTTTCGCGGGGAATACCACACCATC
TTTCATGAAAAGCAGCATACAACGAATTAGGTTTGAAGACAAGCCATCACT
AAAAATTGCAAAAAATGTTAATAGTAACATGGATTACATGCTTTTCTGGAGA
TTGGCAGCATCTCAGCATTACAAGTTCGCCTTTTACAAGAGTGTGGCCAAA
AGACCAGAAGGATGCGTCGATATGAACAATTTACAAGGATCTCCTCACATG
GAGTAG

>g23004.t1 Name:7-deoxyloganetic acid glucosyltransferase
gene=g23004 CDS=1-1443
ATGGACCAACTAAACACCGCAGCGCCGGCGACGGCAGTGGCGGAAGAGC
CACTTCCGCCGCCACACGTACTAATATTCCCATTACCCTTGCAAGGCCATGT
GAACTCCATGCTAAAGCTCGCCGAGCTCTGCTGCATTTCCGGCCTCCATGT
GACCGTGCTCCTCTCCGACTACAATCACGGCCGTCTCCTCCGCCACGCCAA
CGTTAAATCCCGGTTTTCTCGGGGAATCCCAGTTCAGAAATCTGTTGGTTTC
ACTGATCGGCTTAGCAAGAAGACGGGAACCGAGGAGGATGAAGTGAAAA
GGAGACCGGTAACTTGTTTAATTATGGACGGAGTATTGAGCTTTGCTGCCAT
TGTTTCAGAAGAAATGGGCATCCCTTTTATTTATTTCAGGACAGTTGGGGCT
TGCTCTTTCTGGGCTAATTTTTCTATCCAAGAAGTCATTGATGCTGATCAAG
TCCCACTTAGAGGAAAAGGTGAAGACAAAGTAAGCTACAGAAATAAAAAA
GAGATGGATTTATTAGTAACTAGTGTTGCAGGAATGGAAGGATTTCTAAG
GAGGCGAGATCTACCGGCATTTTTCAGGGTTGATGATGTAAATGATCCTGGA
TTTCAGACCATCAAAACAGAGACAAGAAGAAGCATAAAAGCCAGGGCTCT
TATACTGAACACTTTTGAAGACTTGGAGGGGCCGATTCTCAATCAGATTAG
GAAACATATTCCCACTTTGTATTCAATTGGTCCCCTCCATGCCCATCTCAAA
GCCCGGTTTGCAGCAAAACAGTCCGCAACAGAGCAGTCAATGAGCCATTC
TGGGAGTTTTTGGGATGAAGACAGGAGCTGTGTAGAGTGGCTGAACTCAC

AGCCTCACAAATCTGTGATCTATGTGAGCTTTGGAAGTGTTACAATAATGAC
GAGGGATGAGCTTTTGGAGTTTTGGTACGGTTTGGTGAACAGTGGCCAGA
GGTTTCTGTGGGTGATGAGGCCTGATTCAATCACTGGAAAAGATGGGGAG
AGTCAAATTCCTGTGGAGTTAGAGGAAGGCACCAAGGCTAGAGGATACAT
GGTGGGATGGGCACCCCAAGAAGAGGTCCTTGCACATCCGGCAATTGGTG
GATTTTTGACTCACAGTGGGTGGAATTCAACTCTAGAGAGCATATTTGCAG
GAGTGCCAATGATTTGCTGGCCTTACTTTGCAGACCAGACAATCAATAGTA
GGTTTGTAGGTGAGGTTTGGAAGCTTGGATTGGACATGAAGGATACTTGTG
ATAGAGCCACAGTTCAGAATATGGTTAGGGACTTGATGGAAGTGAGGAAA
GATGAGTTCTTGAAGAAGGCAGATCATATGGCTAATTTGGCTAGAAAGGCT
ATCAATGATGGTGGATCATCTTATTGTAATTTGGATAGCCTAATTGAATTTATT
AGATCAATGATTATGTGA

>g30911.t1 Name: 7-deoxyloganic acid 7-hydroxylase gene=g30911 CDS=1-1245
ATGGGAGTGAACTTCAGCTCAGTTGCAATCTTGGGTTTCATTTGTCTTGCCA
TTTACTGGTTTTATAGAGTTTTGGACTGGGCATGGTTAAGGCCTAAGAAGCT
TGAGAAATGCCTGAGGGAACAAGGTTTCAAGGGGAACCCATACAGGCCAT
TTTTGGGTGACCAGTATGAAAGTGGCAAATTAATCAGGGAAGCTATGTCCA
AGCCTATTGGGGTTGAAGAAGATGTCAAGAAACGCATCATACCTCATATCCT
CAAAACTGTCCAAACTCATGGGAAGAATTCTTTCATGTGGGTAGGAAGAAT
TCCAAGGGTGCATGTGACAGATCCAGAACTTATAAGAGAAGTCTTGACCAA
GTACTACAAATTTCAGAAGAATCATCATGATCTTGATCCAATTACCAAATTT
CTATTGACTGGAATAGGCAGCTTAGAGGGGGATCCATGGTCCAGGCGTAGA
AAAATCATCAACTCTGCTTTCCAGTTTGAGAAGTTAAAGCTTATGCTGCCTG
CATTTTACTTGAGCTGTCGTGATATGGTAAGCAAATGGGATAAAAAGGTTCC
TGAGGGTGGTTCAGCAGAGCTGGAAGTATGGCATGATATTGAAACCTTAAC
AGGAGATGTAATTGCAAGAACTCTATTTGGAAGTAACTATGAAGAAGGAAA
AAGAATATTTGAGCTTATTAAAGAACTTACCTCTCTTACCATAGACGTCATT
CGCTCAGTTTACATTCCAGGACAGAGGTTTCTGCCGACGAAGAGGAACAA
CAGGATGAGAGCAATAGACAAAGAAGTAAGAGTTAGGATAACTGAGATCA
TTAATAAGAAAATGAAGGCAATGAAGAAAGGAGAAGCTACTGGTGATAAC
TTCTTGGGCATATTATTGGAATGCAATCTCAATGAAATAAAGGAACATGGGA
ACAACAAAAATGCTGGAATGAGCATCGAAGACATTATTGGTGAATGCAAAC
TATTCTATTTTGCTGGACAGGACACTACCTCAACTTTGATTGTGTGGACAAT
GGTCCTCCTGAGTAGATTCCCAGAGTGGCAGCAACGTGCTAGAGAAGAAG
TGTTGCAAGTTTTTGGGGACAGAAAACCAGACTATGATGGCATAAGCCACC
TAAAAATTGTAACCATGATCTTCAAACGTGATTCAAGCCTTAAGCAGTACTT
AGACAAGCAAAATTGGACACTATTGTACAAGAAGGCAGGTGGAAATGGCC
TGTTGGAGGGATTCTGA

>g43529.t1 Name:Secologanin synthase gene=g43529 CDS=1-1566
ATGGAGATGGATATTACCCATCAGGCCATTGCTGCCACTTGCTTTCTTGCAC
TTTTTGCTTGGGCATGGAGAGTCCTGAACTGGGCTTGGTTTAAACCTAAGA
GGATCGAGAAGCGTCTCAGACAACAAGGTTTCAGAGGGAACCCTTATAGA

TTCTTGGTTGGAGATGTTAAAGAGAGCGGCATGATGCTCCAGGAAGCCATG
TCTAAGCCCATCCCACTCAACAACGATATCGTTCCTCGAATCATGCCTCACA
TTGACAAAACCATCAGGACCTATGGTAAAAATTCCTTTACCTGGATGGGAC
GTATTCCTAGGATCCACGTGATGGATCCCGATCTCATTAGGGAAGTCTTGAC
TCACTCAAACAAATTCATGAAGAATTTTGATGTCCACAATCCACTCGTCAA
GTTCTTACTCACTGGAGTCGGAAGCTTTGAGGGTGAAAAATGGTCCAGACA
TAGAAGAATTATTTCCCCAGCCTTCACTCTTGAGAAGTTGAAGACCATGTT
GCCTGCTTTTGCGGTTAGCTATGACGATCTTTTGAGGAAATGGGGGCAAAC
TGCCACAAGGGAGGGATCCGTTGAGGTGGATATCTTCCCAACATTTGATGT
CTTAACAAGTGATGTGATTTCCAAGGTTGCCTTTGGTAGCACCTATGAGGA
AGGTGGCAAAATCTTCCAACTGCTTAAAGAACTTATGGAGCTCACAATTGA
GACCATGAGAGATGTATACATTCCAGGATTGAGTTTATTGCCAACAAAGAG
GAACAAGAGGATGAAAGAAATTAACAAGGAGATCACAGATATGTTGAGGA
ACATCATCAGCAAGAGAGTTAAGGCAATGAAGGCTGGAGAACCAAGTGAG
GATGATTTACTTGGTGTTCTTTTGGAATCCAACTACCGAGAAATTCAGAAGC
AAGGAAACAACAATAATGTTGGAATGACTATTGATGATGTCATTGAGGAAT
GCAAACTTTTCTACTTTGCCGGACAAGAAACCACAGGTGTCCTTCTCACTT
GGACAACCATTCTATTGAGCAAGCACCCCGAGTGGCAAGAGCGTGCAAGA
GAAGAAGTTATGCAAGCCTTCGGCAAAAACAAGCCTGAATTTGAAAAAT
TGAATCACCTCAAATATGTTTCAATGATTTTGTACGAGGTCCTAAGGCTGTA
CCCGCCAGTCATCGACCTGACCAAAATTGTTCACGAAGATACCAAGCTAGG
ACCATACACCGTCCCTGCTGGAACCCAAATTATGTTGCCAACAGTAATTCTT
CACAGGGAGAAGAGCATTTGGGGAGAAGATGCAATGGAATTCAATCCGGG
AAGGTTTGCAGATGGAGTTGCTAATGCAACCAAGAATCAAGTCACGTATCT
GCCCTTCAGCTGGGGACCTCGCGTCTGCCTAGGCCAGAATTTTGCACTATT
GCAGGCTAAATTGGGGCTGGCAATGATCTTACAGCGGTTCTCCTTCAATGTT
TCGCCATCCTATGCTCATGCTCCTTACACCATCCTCACTCTTCAACCCCAAC
TTGGTTCTCATGTGATCTACCGCAAGCTCGAGTAA

>g2588.t1 Name:Strictosidine-β-D-glucosidase gene=g2588 CDS=1-1530
ATGTCAACTCCAGCTACTGAGTTTTCAGGACCCCTCTCCCGCAGGGACTTC
CCAGAGGGTTTTCTCTTTGGGTCTGCTTCATCTGCCTTTCAGTACGAAGGG
GCTCATAATGTTGATGGCAGACTGCCAAGCATTTGGGATACTTTCCTGACGG
AAACCCATCCTGATATAGTAGCCGCAAATGGACTGGATGCAGTTGAATTTTA
CTACCGCTATAAGGAAGACATAAAAGCCATGAAGGATATAGGGCTTGACAC
TTTCAGATTCTCCCTCTCATGGCCAAGAATTTTGCCCAAAGGAAGAAGAAC
CAGGGGGCCCAACAATGAAGAGCAAGGAGTCAATAAGTTGGCCATAGATT
TCTATAATAAGGTCATTAACTTGCTCCTGGAGAATGGTATAGAGCCTAGTGT
GACCCTCTTCCACTGGGATTTTCCTCAAGCATTGGAAACTGAATACCTGGG
GTTCTTAAGCGAGAAATCCGTGGCGGATTTTGTGGACTATGCAGATCTTTGC
TTTAGAGAATTTGGTGATAGAGTCAAGTACTGGATGACATTCAATGAGACAT
GGAGCTATAGTTTGGCTGGATATCTGATAGGCAATTTTGCTCCTGGCCGAGG
TAGCACTAATGAAGCGCAAAGGCAAGCCATGGTGGAAGTCATCCCATCAA

GTCTAGGAAGTTCGAGGCAGGCATTTGCACACAGTCGCACACCAAGAGCC
GGTGACCCATCCACTGAACCATATATTGTGACACACAACCAGCTTCTTGCTC
ATGCAGCTGCAGTAAAGTTATACAGAACAGCATATCAGAATGCTCAAAAGG
GTAAAATCGGGATTGGACTGGTTACCATTTGGGCTGAACCACACAATGATA
CACAAGAGGACAGAGATGCTGCACAACGAGTGCTTGACTTCATGCTTGGA
TGGTTATTTGATCCCGTGGTCTTCGGCAAGTATCCTGAAAGCATGAGAAGG
CTTTTAGGAAATCGACTTCCGGAGTTCAAACCACACCAACTTAGAGACTTG
ATCGGGTCATTTGATTTTATTGGGATGAACTATTACACTACCAATAGTGTAGC
CAACCTTCCATATTCAAGATCCATCATATATAATCCAGATTCTCATGCTATATG
TTACCCTATGGGTGTGGAGGCAGGCTCTTCTTGGGTTTATAGTTATCCTCAG
GGATTGCTAAAACTGTTGCTCTATGTAAAGGAGAAGTATAATAATCCCCTTA
TCTACATCACGGAAAATGGGATTGATGAGATAAATGATGAAAAATTGACCT
TGTGGGAAGCCCTTTATGATACCCAAAGGATATCCTACCACAAGCAACATCT
TGAAGCCACCAAACAGGCTATCTCACAAGGAGCTGATGTGAGAGGGTACT
ACGCATGGTCGTTTACGGACAATTTAGAATGGGCTTCGGGTTTTGATTCTCG
CTTTGGTTTGAACTACGTGCATTTTGGTAGAGAATTGGAGAGGTATCCAAA
ACTATCTGCTGGCTGGTTCAAGTTCTTCCTCGAAAAGTGA

>g3541.t1 Name:Strictosidine synthase gene=g3541 CDS=1-1122
ATGCCAATGAACTCAAAGCTAATCTTGACTGCTACAGCACTTGCAGTCCTAT
CAATCATCTGGGCTTTCAGCACAACCAATATATTAAGACCACCTCCGATAAG
GGGCTCCCAAGATTTGCTGCACACAGCTGAAACAATTAATCTAACAGGTGC
AGTTGGGCCAGAGAGTCTTGCCTTTGACCCCAGTGGAAAAGGTCCCTATAC
TGGTGTTGCCGATGGTCGGATTCTCAAGTGGCAAGGACATCAACATGGTTG
GGTTGATTTTGCATTCACCTCTTCTCAAAGGAAGGAGTGTGTTCATCCATTT
GCACCACATATGGAGCATATATGCGGAAGACCATTGGGCCTTCGATTTGATC
AGAAGAATGGAGATCTCTATATTGCTGATGCCTATTTGGGCCTCCAAGTGGT
AGGGCAAATGGGAGGTTTAGCAACTCCACTAGTGACAGAGGTCGAAGGTG
CTATTTTGCGTTTTACAAATGACATGGACATTGATGAGCATGAAGATGTGAT
TTACTTCTCAGATACAAGCATGATATTCCAACGAAGGCAATTTATATCAGCC
GTCTTAAGTGAAGACAAGACAGGCAGACTGATGAAATATGATAAATCCAGC
AAAGAAGTAACAGTTCTACTTCGGGGACTTGCTTTTGCTAATGGTGTGGCT
TTGAGTAAAGACCGCTCTTTTGTGCTTGTGGCTGAAACCACTGCTTGTAGA
ATAGTGAGATTCTGGCTTCGAGGCCCTAATGCCGGAAAACATGATATCTT
TGCTGAGCTGCCAGGATTTCCTGACAACATTAGAAGGAATTCGAAAGGGG
AGTTTTGGGTGGCTTTGCATTCAAAGACTGGACTTCTTTTAAAGCGGGTGA
TTTCTAATTCTTGGTTTGGAAAGACTTTGCTAAAGCTTCCGCTAAGCTTCAA
GCAACTGCACCATTTTTTAGTGGGAGGGCAGGCTCATGCAACCGCAATAAA
ACTGAGTGAGGAGGGGGAAGTTTTGGAGGTCTTGGAAGATATTGAAGGGA
AGACCTTGAGGTTTATAAGTGAAGTTGAGGAGAAAGATGGGAAGTTATGGA
TTGGATCTGTTTTGATGCCTTTTATTGGAATTTACAAATTGAACTAA

>g8946.t1 Name:Anthranilate synthase gene=g8946 CDS=1-1728
ATGGAATCCCTAGCTCTGTCTCATCGGCCACTTCCCTCCATTTCCTCCTGTTT

CAAAACCATCTCAATTACCACCTCCAAAACCCCGCGTTTCCTACGTACATTT
AAATGCTCCACCATTTCTCCTCCATCATTAGTGGACCAGTCGGCGAAGTTTA
AGGAGGCCGCGAAACATGGGAATTTGATCCCTTTATACAGGTCTATATTCTC
CGATCACCTGACCCCTGTGTTGGCTTACAGGTGTTTGGTCAAGGAAGATGA
CAGAGAGGCTCCCAGCTTTCTATTTGAGTCTGTTGAACCCGGTTTGAAAGA
CTCCAGCGTAGGGCGATATAGTGTGATTGGAGCTCAGCCAACAATGGAAAT
TGTAGCAAAACAGAACATGGTAACCGTAATGGATCATCGTCAAGGACGAA
GGGTGGAACAGTTTGAAGAGGATCCAATGGTTGTTCCTCGGAGGATTATGG
AGAAATGGAAAACACAGCGCATTGAGGAGCTCCCTGATGCATTTTGTGGTG
GTTGGGTTGGTTATTTTTCATATGACTCGGTGCGCTATGTTGAGAAGAAGAA
GCTTCCCTTCTCAAATGCTCCTATCGATGATAGAAACCTTCCTGATGTTCATC
TAGGTCTTTATGATGATGTAATTGTTTTTGATCACGTGGAGAAGAAAGCGTA
TTTGATTCACTGGGTGCGGTTGGATGAATTTTCTTCCGTGGAGGAGGCCTA
CAATGACGGAATCAATCGGTTGGAGTCTTTGGTGTCTAGAGTGCATGATATA
ATCCCTCCCAGGCTAGCTGCAGGTTCAGTTAACTTGTATACAAGCCTTTTTG
GTCCTTCATTAAAGAAGTCAACCTTGACAATCGAAGACTACGAGAAGGCA
GTGTTGCAAGCCAAGGAGCATATCTTGGCTGGGGACATTTTCCAGATTGTT
CTAAGTCAGCGTTTTGAGCGACGAACATTTGCAGATCCATTTGAAGTTTAC
AGAGCATTAAGAATTGTCAATCCCAGTCCATACATGACTTATTTGCAGGCAA
GAGGATGTATTCTGGTTGCATCAAGTCCTGAAATTCTTACCCGAGTTAAGAA
GGGAACAATAACTAATAGGCCATTAGCTGGGACTACTAGGAGGGGAAAGA
CACCCAAAGAAGACTACATGTTAGAAACTCAGCTCCTTCATGATGAAAAAC
AGTGTGCAGAACACATTATGTTGGTTGACTTGGGAAGGAATGATGTTGGAA
AGGTGTCAAAACCTGGTTCTGTGAATGTTGAGAAACTTATGAACATTGAAC
GCTATTCCCATGTCATGCACATCAGCTCCACGGTTACTGGAGAGTTACTTGA
TCATTTAACTAGCTGGGACGTTCTTCGTGCAGCGCTGCCTGTTGGGACTGTT
AGTGGAGCCCCAAAGGTAAAGGCCATGGAATTGATCGATCAGCTGGAAGT
CACGAGGCGTGGGCCTTATAGTGGAGGATTTGGAGGAATTTCTTTTTCCGG
TGATATGGATGTTGCACTAGCTTTGAGAACAATTGTTTTCCCTACTGGAACA
CGTTATGACACCATGTACTCTTACAAGGATGAGATCAAACGCAGAGAATGG
ATTGCCCATCTTCAATCTGGGGCAGGTATAGTAGCTGATAGTGTTCCTTCCG
ATGAGCAGAGAGAGTGTGAAAATAAAGCTGCTGCTCTTGCCCGTGCAATTG
ATCTCGCGGAGTCATCATTTGTTGAGAAATAA

>g21597.t1 Name:Anthranilate phosphoribosyltransferase
gene=g21597 CDS=1-1233
ATGGCCTTGTTTCATCAGTGTCTACCTTTCGCTTCTTCACTTAACTGTGCAAT
TTCAGACCTGAATCTTTCAAGAAAATTTGAGCTAAAAAATTCAATCTTTGTC
AGTAATCCATTTGGGGAAACAGCTCAAAGAAGGCCCCAAAATGTTGTCTTA
GCGCTGCCCACTTCTCATAAGGTTTCATCATCCATATCTTCTTTCGGTCAGTT
GATTGAGACGTTGATAAGTGGGGTTGACTTATCAGAATCTGAAGCTGAGGA
ATCTCTTGATTTTTTGCTGGATGGTGCCGATGAGACATTGATCAGTGCCTTT
CTTGTTTTGTTGAGAGCCAAAGGAGAAACTTATGAAGAAGTGGTAGGATTG
GCAAGGGCAATGATAAAGCATTGTAAGAAAGTGGAAGGATTAGATGATGCT

GTAGACATAGTTGGGACTGGTGGTGATGGTGCAAATACTGTGAACATTTCA
ACTGGTGCTTCTATTCTTGCTGCAGCTTGTGGCGCAAAAGTTGCAAAGCAA
GGAAATAGGTCAAGCTCTTCAGCTTGTGGAAGTGCTGATGTTCTTGAAGAA
TTAGGAATTGCAATTGAGTTGGACCCTGAGGGGGTAAAGAGGTGTGTAAAT
GTAGCTGGAATTGGTTTCATGATGTCTCCAATCTACCATCCAGCCATGAAGA
TTGTTAGGCCAATCAGGAAGAAGCTGAAAGTAAAGACTGTATTTAATATCTT
GGGCCCAATGTTGAATCCAGCAAGAGTACCTTTTGCTGTTGTGGGTGT
ATACAAAGAAGATCTAGTCCATAAAATGGCAAAAGCACTACAAAGATTTGG
AATGAAAAGAGCATTGGTCGTTCACTCTGAAGGCCTGGATGAGATGAGTCC
CCTTGGGCCTGGGCTAGTTCTTGATGTCACTGCAGATAAGATTGAAAAGTT
CTCATTTGATCCTTTGGACTTTGGGATACCTCGGTGCACTTTGGATGGCTTG
CGCGGTGGTGGACCAGAGTACAATGCAGAAGTTCTAAGACGTGTACTATCA
GGCGAGCAAGGGGCCATTGCTGATGCACTTGTGCTAAATGCAGCAGCAGC
CATCTTGGTTAGTGGACTTGTAAAAGATCTGGCTGAAGGAGTGACCTTGGC
TCGAGAAACATTGTTATCAGGGAAAGCAATAAAGACCCTTAATTTGTGGAA
AGATATCTCCAATAAGGCGAAAGAAGAAACACATAGTAACTTGGTATCTGC
ATAA

>g31672.t1 Name:Phosphoribosylanthranilate isomerase   gene=g31672 CDS=1-828
ATGCTATCAGGTTTGAATGTGGGGACGCAGCTCCAACCCAGAGCCACAAG
CATTCCCAGATGGGAATGGGTTCTAGAAAGAAGTGGAATGCATTCATTAAA
CACCAGAAGAACTTGTGCAAGGAATATGACCACATGCTTGCTTAATCAACA
GCCAGTTTTCCCTCCTGATAGGGAACAAAAAGAAAGTAGGCCTTTAGTTAA
AATGTGTGGAGTTACATCAGCTAGAGATGCTGCTCTGGCATCGGAAGCTGG
TGCTGATTTTGTTGGAATGATTCTTTGGCCTAACTCAAGACGTTCTATTCCA
CTTCCTGTTGCCAAGGAAATCTCAAAAGTTGCACGGGAGAATGGAGCAAA
GCCTGTTGGTGTGTTTGTAGACGATAATGCTGACACAATATTAAGAGTTTCT
GAAGCCACTGACCTTGAATTTGTGCAGCTGCATGGAAATGGTTCCCGGATG
GCTTTTCCTATTGTCCGGAGAGAAAACCGTGTAATTTTTGTTCTTCATGCAA
ATGAAGATGGAAGCCTACTGAACTCTATTTCCGATGAAGATTGTTCTCTAGT
TGACTGGATTCTTGTCGACAGTGCCAAAGGAGGAAGTGGCAAGGGTTTTA
ATTGGTCTAGGTTTAAGCTGCCACCAATCACAAGCAAACATGGTTGGCTCT
TAGCTGGAGGGATTAATCCCATGAATGTGTCTGAGGCTCTTTCAGCTCTAAA
ACCTCATGGAGTTGATGTTAGTAGCGGCATTTGTGGTCCTGATGGCATCCAG
AAGGACGAGTCGCTGATGCTGTCTTTCATGAATGCAGTCCGTTTTGTAAAC
CATCAATGA

>g3418.t1 Name:Tryptophan synthase alpha chain   gene=g3418 CDS=1-963

ATGGCTGGTGCTGCTGCCCTGAAAGCTAGCTGTTTTCTCCAGCTGAAGCAG
AAACCTCAGGACCGTTCGTCCCTCCTTCTTCCTCAAAGAATCGGCCTATCA
GCTTCTAATACTCTTGGTAGATCATTCAAGCCTCCTATGGCGACCCTCAGCA
CTGTTCCAGCTGTCGGGCTGTCTGAGACTTTCTCAAAACTGAAAAAACAA
GGCAAAGTGGCTTTTATACCATATATCACTGCCGGCGATCCTGACCTTTCGA

CTACAGCTGAAGCATTGAAAGTGCTTGATGCATGTGGTTCTGACATAATTG
AACTAGGGGTACCTTATTCTGATCCATTGGCTGATGGTCCAGTTATACAGGC
TGCTTCAACACGTGCACTGGCCAGAGGGACAAATTTTGATAAGATTATTTC
CATGTTGAAGGATGTTGTACCTCGGTTATCTTGTCCAGTTGCATTATTCTCAT
ATTATAATCCAATACTTAAGCGTGGTGTTGAAAAGTTTATGATCACCGTCAG
AGATGCTGGGATACATGGACTTGTGGTTCCAGATGTACCTCTGGAGGAGAC
TGAGATCTTGAGAAATGAAGCCACTAAGAGTAATCTTGAATTGGTGTTGCT
GACAACACCGACAACTCCAACAGCTCGAATGAAAGCAATTGCCGAAGCTT
CAGAAGGGTTTGTATACCTCGTGAGCTCGGTTGGAGTTACTGGAGCTCGCG
CATCTGTGAGCAAACACGTGCAATCTCTCCTGACGGACATTAAAGAGGCAA
CAAATAAGCCAGTAGCAGTTGGTTTTGGAATATCGAAACCTGAGCATGTGA
AACAAGTTGCTGGATGGGGAGCAGATGGAGTGATTGTTGGCAGTGCCATG
GTAAGAATACTAGGCGAGGCAAAATCTCCTGAAGAAGGGTTGAAAGAGCT
AGAAATCTTCACAAAGTCACTGAAAGCTGCATTACAGAGATGA

>g26350.t1 Name:Tryptophan decarboxylase gene=g26350 CDS=1-1530
ATGGGCAGCATTGATGCGAGTGATGGTACTGTTTATGCCAATTCAGCAGTTG
CACCATTCAAGCCACTTGATCCTGAAGAATTCAGAAAACAAGCCCATCGTA
TGGTTGATTTCATAGCTGACTATTACAAAAATATTGAAAACTATCCTGTTCTC
AGCCAAGTGGAGCCGGGATATCTCCGAACCCGGCTACCGGAATCTGCCCCT
TATCTCCCTGAGCCATTTGAAAATATTCTGAAAGATATTCAAAAAGATATAAT
CCCTGGAATGACCAACTGGTTGAGCCCCAACTTTTTTGCATATTTTCCTGCT
ACAGTTAGCTCTGCCGGTTTTCTTGGAGAAATGTTGTG
CACCGGCTTTAACTCTGTTGGTTTCAACTGGCTCGCTTCCCCGGCCGCAAC
CGAGCTCGAAATGGTTGTGATGGATTGGTTGGCTAACATGCTTAAGCTCCCT
AAGTCCTTCATGTTCTCTGGCACTGGTGGTGGTGTTCTCCAGGGAACAACC
AGTGAGGCCATTCTTTGCACCATCATCGCCGCCCGCGACGGGGCTTTCGAG
AAAATCGGTGTCGAAAACATCGGAAAGCTTGTTGTCTATGCTTCTGATCAA
ACACACAGTTTTTTCGTCAAGACTTGCAAATTGGCTGGTATTTTCCCGTGCA
ATATAAGAATAATCCCTACCACTGCTGAAGACAACTTTTCCATGTCCCCTGA
GGCTCTGCGTAAACAAATTAAAGCTGACGTGGAAGATGGACTGGTCCCAC
TTTTCATCTGTGCTACTGTGGGGACAACTTCTACCACTGCCATTGACCCGGT
GAGTCAACTAGCCGAAGTTGCTAACGATTTTAAGGTGTGGATTCACGTTGA
TGCTGCTTATGCAGGCAGTGCATGCATATGTCCCGAGTTCAGGCAATACTTG
GAAGGGATTGAGCAAGTTGACTCGTTCAGTTTTAGCCCTCATAAATGGCTA
CTTTGTTATTTGGATTGCTGTTGCCTGTGGGTCAAGAGACCCGAGTTGATGG
TGAAGGCATTAAGCACCAACCCGGAGTATTTGAGAAATAAAAGAAGTGAG
TTTGATTCAGTTGTGGATTTTAAAGATTGGCAGATCGGTACAGGTAGACGTT
TTAGAGCATTACGACTATGGCTTATTATGCGTAGTTATGGAGTTGCAAACCT
ACAAAGCCACATTCGATCTGATGTTCAAATGGCCAAGATGTTTGAAGGCTT
TGTGAAATCTGATGCAAGATTTGAAATAGTAGTACCGCGTGCATTCGCGCTT
GTATGCTTTAGGCTTAACCCTTTGAGAGGATCCAGTCCAACATACTTGGAGC
TTTTAAACAAGAAGCTACTCGAGATAGTCAACTCAACGGGCCGGGCTTACA
TGACTCACACTAAGGTTGGTGG

AGTCTACATGCTCAGATTTGCAGTTGGGGCTACACTAACCGAGGATCGCCA
TGTGTATGCTGCTTGGGAG
TTGATTAAAGAATGCGCTGACGCTTTACTTAAAGAGAAAATCTTTATGACTG
ATGAATAG

>g2990.t1 Name:WRKY1 gene=g2990 CDS=1-1689
ATGTCAAGTGATGTGCAGCCAAGAGAGAGCCTGCATAATGGGGTTCCTGTG
TCACAATCTGATCAAGAAGAAATTGCTTCCTCTGTCTTGCCAAAGAGTGAA
TCAGGAATTCTTCATCAAAAACTGAGCTCTGCAAGTGAAGGATGTGAGTCA
GAGGTACATAAATTACCTACTCTAATAAAAAAAGAGCAAGATGACATGAAT
CAGAGACATAGCCCTGATACACAAAGTGATTCAGTGGCATGTGGTGAAACA
GAGACACCTGATAGCGGAAGCCATGCAACAATGTCCAACAAAGTTTCTCTA
GAACCCAAAAAGGAGCTAGTTCATGATGGCGGGGTTTATGCATCAGTGTCC
AGTAAGAAACCAATTCCTGAGAATGGAGACCCTGCATCGCAGTTCAATCAA
TCGCCTGTGCTACCAAAGAAAGAGCTGGATAATGTGGAGCAGAGTATGAGT
TCAGATGCTACAATCAATCTATCATCATGTGCTGAGAAACAGGTACCTGTTG
GGGTCCACGTGTCACAGTTCAATCAAGTTTCTATTATCGCGAAAGTGGACC
CAGACAGTCTGGTGCAGGGACAAAGCCCTGATGCTGGAAGAAATGCATCA
GCACCCCATGAAAGAGGAAGAACTGACTCCATAATGCTTGAGAAGTTTTCA
CAACGTATGCGAAGTACTACCCAGCAGATGTGCACATCGTCATCTGATCAG
GAGAAAATGACTTTTTCTGGTAGATCAGAGAAAATTTTGTACAAGTTGCAG
CCAAGACGAAACCCTGATACTGGGATCCATACATCGCAATCTGAAAAAGGA
ATCACTTCCCCTAGAATACGTGAAAAAGCATTGGATGATGGATACAATTGGC
GAAAATATGGACAGAAGCTTGTTAAAGGAAATGTTTTTGTCAGGAGCTACT
ACAAATGCACTTACTCTAATTGTCGAGCAAAAAAGCAGGTGGAGAGATCC
CATGATGGACATCTTACTGATATTAAGTACATTGGAAAACATGAACATCCTA
AACCTCAAAGTAGTCCCAAAGATACTGCATTTGTCTCACCTCCCGAAATGA
GTAAAGCAGACATTCTTGTTACAGCTACATCACAAGCTGAAGATGAGCCAT
CTGCTGGACCTGGGGATATATCTCAACCTAGTGCGCCAACAGAAACCCCAC
GGGAGCTGATTGCCACAGCAAGTAACCATAGCAAGGGAGGGGCAGTTTCA
CAGTTGCATAATTCAAAAGATGATATTGACAGTGACATATCCAAGAGACAG
AAGAGAGAAACTTGTAAGGTTGATGATAATCAAGTGAAAAGGACTTACTGT
GAATCACGGCAAGTAGTTCAAATGATGAGTGAGGTTGACATAGTAAATGAT
GGCTATCGCTGGCGCAAATATGGGCAGAAATTGGTTAAAGGAAATCCCAAT
CCGAGGAGTTACTACAGATGCTCAAATGCTGGTTGCACGGTCAAGAAACAT
GTTGAGAGGGCATCCCATGATGCAAAAGTAGTCATTACAACATACGAAGGA
AAGCACGATCATGATATGCCTGCTTCTAGAACTGTTGTCAATAGTACAGCAG
AGACAAGATCTGAGCTTGGAGAACACGAACCTTTTGGTGTGGATATGGTCT
TCTATGACCAGCTGATCGAAGAGTGGGTTAGGGTGGTGGGTGGTGAAAGA
GGTTGA
